# Twin Embryos in *Arabidopsis thaliana KATANIN 1* Mutants

**DOI:** 10.3390/plants13131824

**Published:** 2024-07-03

**Authors:** Youfeng Yu, Rui Zhu, Hao Xu, Balaji Enugutti, Kay Schneitz, Xuanpeng Wang, Jing Li

**Affiliations:** 1 College of Life Science and Technology, Huazhong Agricultural University, Wuhan 430070, China; 2 Sanya Institute of Breeding and Multiplication, Hainan University, Sanya 572025, China; 3 School of Tropical Agriculture and Forestry, Hainan University, Haikou 570228, China; 4 Plant Developmental Biology, TUM School of Life Sciences, Technical University of Munich, 85354 Freising, Germany; balaji@mytum.de (B.E.);

**Keywords:** *Arabidopsis thaliana*, *KATANIN 1*, microtubule, twin embryo, embryogenesis

## Abstract

Regulation of microtubule dynamics is crucial during key developmental transitions such as gametogenesis, fertilization, embryogenesis, and seed formation, where cells undergo rapid changes in shape and function. In plants, katanin plays an essential role in microtubule dynamics. This study investigates two seed developmental mutants in *Arabidopsis thaliana*, named *elk5-1D* (*erecta-like 5*, *ELK5*) and *loo1* (*lollipop 1*), which are characterized by round seeds, dwarfism, and fertility defects. Notably, *elk5-1D* exhibits a dominant inheritance pattern, whereas *loo1* is recessive. Through positional cloning, we identified both mutants as new alleles of the *KATANIN 1* (*KTN1*) gene, which encodes a microtubule-severing enzyme critical for cell division and morphology. Mutations in *KTN1* disrupt embryo cell division and lead to the emergence of a twin embryo phenotype. Our findings underscore the essential role of KTN1 in fertility and early embryonic development, potentially influencing the fate of reproductive cells.

## 1. Introduction

In angiosperms, seed development occurs through the double fertilization of the ovule, resulting in the formation of three main components: the embryo, endosperm, and seed coat. The embryo and endosperm are generated by the fertilization of the egg cell and central cell by sperm, while the seed coat originates from the integument [1]. In *Arabidopsis thaliana*, prior to fertilization, the polarity of the egg cell along the apical-basal axis is established by an asymmetric arrangement of organelles and microtubules orientated longitudinally [2,3]. Following fertilization, the zygotic cell, which expands along the same axis, divides asymmetrically, creating two distinct cells. The smaller apical cell develops primarily into the proembryo, and the larger basal cell gives rise to the suspensor [4].

High-order cell division plays an essential role in the development of seeds. This ordered process of cell division not only influences the size and shape of the seed but also directly affects the nutrient content and the normal development of the plant embryo [5]. Microtubules play a critical role in the cell division process, where the symmetry or asymmetry of cell division is primarily determined by the symmetry of the preprophase microtubule band (PMB). The PMB is a cortical microtubule ring formed during the interphase by reorganizing the diffuse cytoplasmic microtubule system. This ring gradually narrows and disassembles simultaneously with the nuclear envelope at the beginning of mitosis [6,7]. Before the complete disassembly of the PMB, microtubules rapidly aggregate to form the spindle apparatus. The spindle microtubules are arranged in a dense array, exerting sufficient tension to facilitate chromosome separation during cell division [8,9]. Subsequently, a phragmoplast appears between the two sets of chromosomes, generating a new cell wall and resulting in the separation of two daughter cells [10]. Moreover, the morphology and directional growth of plant cells are predominantly governed by the deposition of cellulose microfibrils within the cell wall, which is intricately regulated by cortical microtubules [11,12].

The dynamics of microtubules, regulated by severing proteins such as katanin, are essential for cell division and differentiation [10,13]. Katanin belongs to the AAA-ATPase family with microtubule severing function. In mammalian cells, in addition to katanin [14], the family also includes spastin [15] and fidgetin [16,17], while in plants, only katanin has been identified [18,19]. Mammalian katanin is assembled by a catalytic subunit of approximately 60 kDa (p60) and a regulatory subunit of 80 kDa (p80) [20]. Katanin forms a hexameric ring on the microtubule surface and utilizes ATP hydrolysis to catalyze microtubule severing [21]. In *Arabidopsis thaliana* genome, there is only one gene encoding the p60 subunit, and four genes are responsible for the p80 subunit [22,23]. In vitro studies confirm that the p60 subunit KTN1 exhibits microtubule-severing activity [24]. Within the cell, KTN1 primarily severs newly formed microtubules from pre-existing microtubules and those that intersect during dynamic changes [25,26,27].

In this study, we describe the characterization of *elk5-1D* (*erecta-like 5*, *ELK5*) and *loo1* (*lollipop 1*) mutants in *Arabidopsis* that exhibit round seeds, a dwarf phenotype, and fertility defects. Through the application of positional cloning techniques, we identified *elk5-1D* and *loo1* as new alleles of the *KTN1* gene. The *elk5-1D* is defined as a dominant mutation caused by a base substitution that alters the second-last amino acid residue at the C terminus of KTN1. Meanwhile, the *loo1* mutant involves a recessive mutation caused by the deletion of a small fragment, which leads to the formation of a premature stop codon. In *Arabidopsis*, the *ktn1* mutants reportedly exhibit pleiotropic phenotypes, affecting almost all vegetative and reproductive organs [13,18,19,23,24,28,29,30,31]. In the *elk5-1D* and *loo1* mutants, the seed sizes are notably larger with a distinct round shape. To understand the underlying reasons for these phenotypic alterations in seed development, this study focused on observing the initial developmental stages of these mutant seeds. We noticed irregularities in embryo growth, particularly the phenomenon of twin embryos emerging. These results indicate that *KTN1* is crucial in controlling cell fate during the reproductive process.

## 2. Results

### 2.1. Identification and Phenotypic Characterization of Two Round Seed Mutants

**Figure 1:**
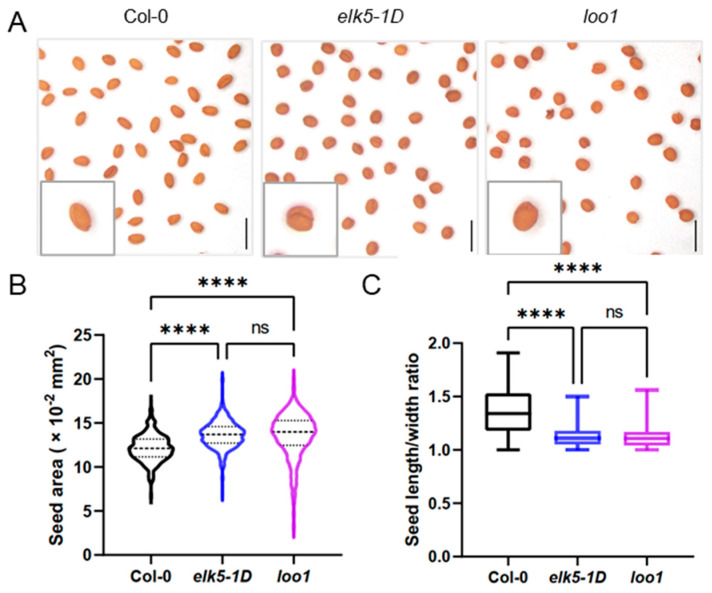
Abnormal seed development in *elk5-1D* (*erecta-like 5*, *ELK5*) and *loo1* (*lollipop 1*) mutants. (**A**) Representative images of mature seeds in Col-0 and *elk5-1D* and *loo1* mutants. Scale bars = 0.5 mm. (**B**,**C**) Quantitative comparisons of seed area and of seed length/width ratio among Col-0, *elk5-1D*, and *loo1*. Final calculations were based on data collection from 443 to 457 seeds. Asterisks indicate significant differences compared to Col-0. Asterisks indicate significant differences analyzed by one-way ANOVA tests (ns: not significant *p* > 0.05, ****: *p* < 0.0001).

The *elk5-1D* (*erecta-like 5*, *ELK5*), exhibiting an erecta-like phenotype with flowers clustered at the inflorescence apex, was obtained from TAIR (The Arabidopsis Information Resource). The morphology of the *elk5-1D* mutants is reminiscent of the *erecta* (*er*) mutant; however, the heterozygote *elk5-1D* mutants display an intermediate phenotype between wild-type and *elk5-1D* (https://abrc.osu.edu/stocks/36011 (accessed on 4 May 2022)). In addition to the erecta-like phenotype, we found that the *elk5-1D* mutant also exhibits a distinctive round seed shape (Figure 1A). Simultaneously, another round seed mutant, *lollipop 1* (*loo1*), was characterized with a phenotypic profile identical to that of *elk5-1D*, with the exception that it is recessive. By quantifying the seed area, it was determined that the seed size in round seed mutants is markedly greater than that found in the wild type. Furthermore, we calculate the length-to-width ratio of the seeds and observe that, in contrast to the wild type, the round seed mutants have a ratio closer to 1, indicating a more spherical shape (Figure 1B,C).

### 2.2. Decline in Fertility in elk5-1D and loo1 Mutants

**Figure 2:**
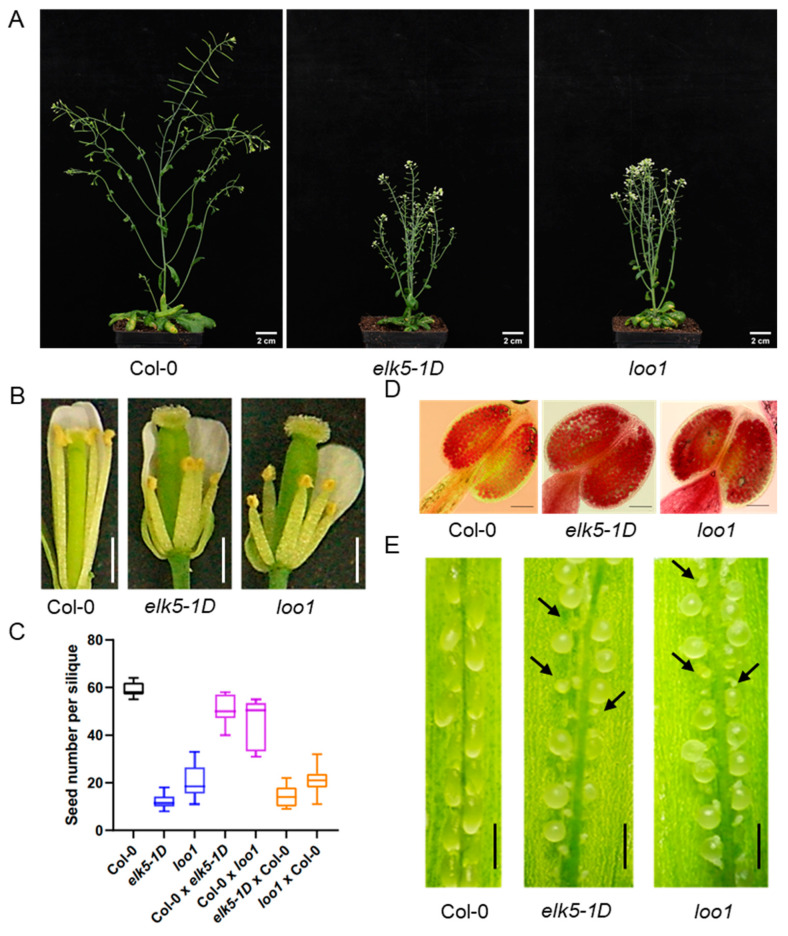
Observation of *elk5-1D* and *loo1* fertility. (**A**) Morphology of 8-week-old plants. The main inflorescence stem of *elk5-1D* and *loo1* (**middle** and **right**) is much shorter than that of wild-type (**left**). Scale bars = 2 cm. (**B**) Representative images of flowers of Col-0, *elk5-1D*, and *loo1*. Scale bars = 0.5 mm. (**C**) Number of seeds per silique in Col-0, *elk5-1D*, *loo1*, and reciprocal crosses, with *elk5-1D* and *loo1* subjected to artificial pollination. (**D**) Mature pollen grains were stained with Alexander solution; viable pollen appeared purple. Scale bars = 0.2 mm. (**E**) Wild-type siliques showing a complete seed set, while siliques of *elk5-1D* and *loo1* plants subjected to artificial pollination show many unfertilized ovules (indicated by black arrows). Scale bars = 0.5 mm.

These round seed mutants display reduced fertility as evidenced by the presence of numerous non-elongated siliques on the *elk5-1D* and *loo1* inflorescences, indicating that the irregular development of siliques could be a result of fertilization defects (Figure 2A). Further investigation revealed that the stamen length in the mutants was significantly shorter than the pistil length (Figure 2B). In particular, both mutants (*elk5-1D* and *loo1*) did not show a significant difference in pollen vitality compared to the wild type, as demonstrated by Alexander staining of pollen (Figure 2D). This suggests that fertility problems are likely not attributed to pollen viability. Meanwhile, the number of seeds per silique of *elk5-1D* and *loo1* significantly increased with artificial pollination compared to self-pollination, but remained significantly lower than that of the wild type (Figure 2C). The widespread presence of residual aborted ovules in artificially pollinated siliques suggests that failed fertilization is probably responsible for the reduced seed count in mutants (Figure 2E). To further investigate the causes of the fertilization defects, reciprocal crosses were conducted between the wild-type and mutants. To eliminate the impact of shortened stamens, artificial pollination was used in our investigation of reciprocal crosses between wild-type and mutants. When mutants were used as male parents in crosses with the wild type, the seed number per silique significantly increased, albeit slightly less than in wild-type self-pollination, indicating a minor pollen defect. However, when these mutants served as the female parent, the number of seeds per silique was significantly lower than that of the wild type (Figure 2C). These findings imply that fertility defects in round seed mutants are primarily attributable to the developmental defect in female gametes, along with the reduction in stamen length.

### 2.3. Phenotypic Alterations in the Morphology of elk5-1D and loo1 Mutants

**Figure 3:**
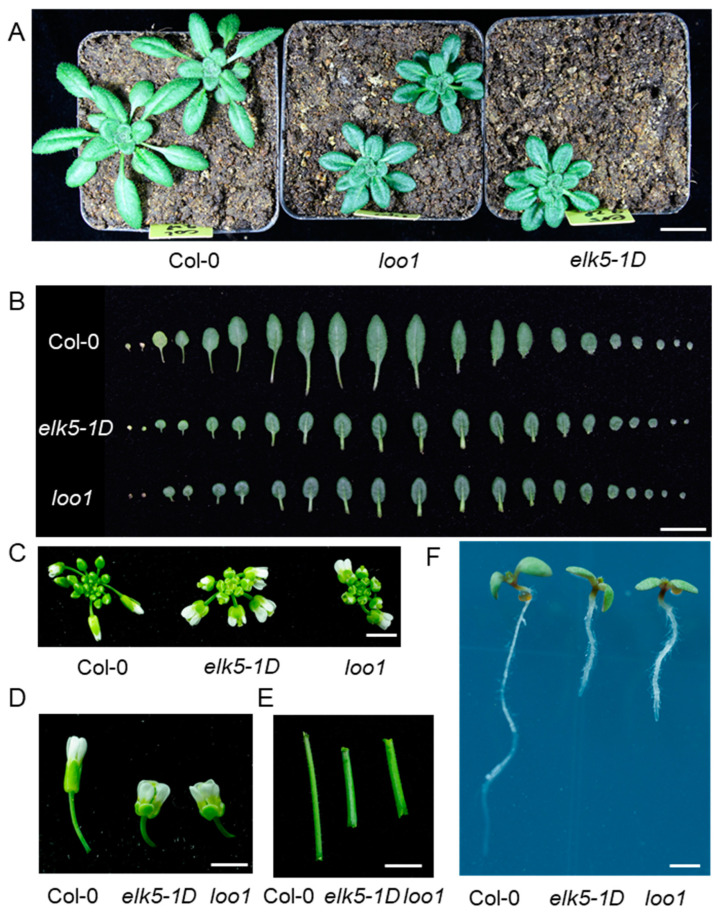
Morphologies of the *elk5-1D* and *loo1* mutants. The phenotypes of rosette leaves (**A**), individual leaves (**B**), inflorescences (**C**), flowers (**D**), main stems (**E**), and root lengths (**F**) among Col-0, *elk5-1D*, and *loo1*. The scale bars measure 2 cm in (**A**,**B**) and 0.5 cm in (**C**–**F**).

In addition to fertility defects, the *elk5-1D* and *loo1* mutants exhibited a dramatic alteration in plant morphology. The height of these mutants was approximately half that of the wild type (Figure 2A). The leaves of mutant plants were much shorter than those of the wild type, resulting in more compact rosette leaves compared to those of the wild type (Figure 3A,B). Meanwhile, the floral organs of the *elk5-1D* and *loo1* mutants were markedly shorter, giving the inflorescence a more compact appearance (Figure 3C,D). Furthermore, the main stems of these mutants were noticeably thicker than those found in the wild-type plant (Figure 3E). Lastly, the root length of the *elk5-1D* and *loo1* mutants was also reduced compared to the wild type (Figure 3F), underscoring the extensive nature of the morphological deviations observed.

### 2.4. Positional Cloning of the elk5-1D and loo1 Gene

**Figure 4:**
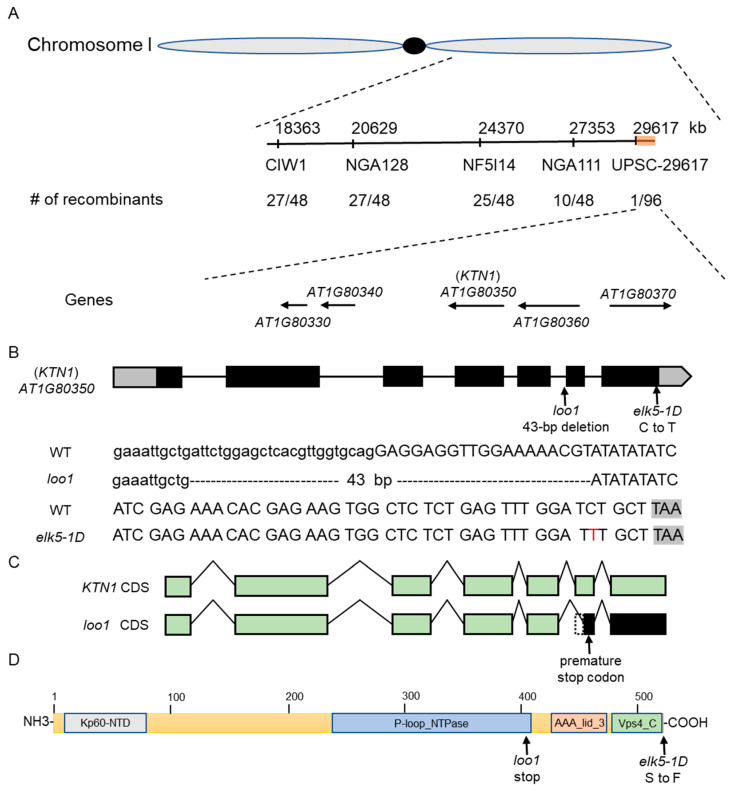
Diagram of the position of the *elk5-1D* and *loo1* gene. (**A**) Fine mapping of the *elk5-1D* and *loo1*. The numbers below the markers indicate the number of recombinants detected between the markers. (**B**) Gene structure of *KTN1* and the location of SNPs in mutant *elk5-1D* and the site of deletions in mutant *loo1*. (**C**) *ktn1* transcript in *loo1* detected by RT-PCR. (**D**) A schematic diagram of the KTN1 protein, indicating the Kp60-NTD, P-loop_NTPase, AAA_lid_3, and Vps4_C (oligomerization domain) dimer domains, is indicated in the bottom panel. The mutation positions of the *elk5-1D* and *loo1* mutants are shown.

To clone the *elk5-1D* and *loo1* gene, we generated a mapping population from the cross *elk5-1D* (Col-0) × Ler and *loo1* (Col-0) × Ler, and identified 600 and 550 homozygous mutants in the F2 generation, respectively. Initially, we utilized SSR markers to genotype the homozygous mutants, successfully identifying the mutation of *elk5-1D* and *loo1* within chromosome I. Subsequently, we selected five SSLP markers for the fine mapping of the mutant gene. This strategic approach allowed us to precisely narrow down the location of the *elk5-1D* locus to a confined 810 kb genetic window located between Upsc-29617 and the terminal region of chromosome 1 (Figure 4A). In particular, the *loo1* locus was also positioned within this same region. Among 236 genes in this region, we noticed *AT1G80350* encoding KTN1, a protein with a microtubule-severing function. *KTN1* mutations were reported to have phenotypes identical to those of *elk5-1D* and *loo1* [13,18,19,23,28,30]. Therefore, we sequenced the *KTN1* gene in the *elk5-1D* and *loo1* mutants and identified mutations in both alleles (Figure 4B). Specifically, *elk5-1D* harbors a substitution of C to T nucleotides in the seventh exon, altering the encoded amino acid from serine to phenylalanine at the last second amino acid residue. Meanwhile, the *loo1* mutant harbors a 43 bp deletion bridging the fifth intron and the sixth exon. This deletion may result from chromosomal recombination following the insertion of 4× 35S enhancers. Consequently, the fragment deletion leads to a premature termination code, producing a C-terminal truncated version of KTN1 (Figure 4C,D). Additionally, we conducted RNA sequencing on developing *elk5-1D* seeds and found no other meaningful mutations other than those in *KTN1*.

### 2.5. elk5-1D Is a Dominant Mutant

**Figure 5:**
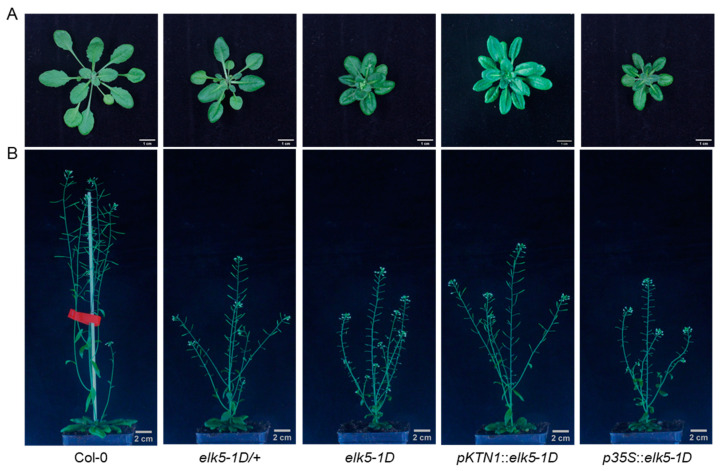
Rosette leaves and fertility phenotypes of plants that harbor the *elk5-1D* allele. Representative images of rosette leaves (**A**) and fertility phenotypes (**B**) from wild-type Col-0, heterozygous *elk5-1D* mutants, homozygous *elk5-1D* mutants, and transgenic plants harboring the *pKTN1*::*elk5-1D* and *p35S*::*elk5-1D* constructs. The scale bars measure 1 cm in (**A**) and 2 cm in (**B**).

The *elk5-1D* heterozygous mutant shows rosette leaves similar to those of *elk5-1D*, along with partial restoration of fertility (Figure 5). To establish *elk5-1D* as a dominant mutated allele of *ktn1*, we used native and 35S promoters for the expression of the mutated *elk5-1D* allele and subsequently introduced it into the wild-type Col-0. The rosette leaf morphology of transgenic plants carrying *pKTN1*::*elk5-1D* or *p35S*::*elk5-1D* is nearly identical to that of *elk5-1D* mutants (Figure 5A). The fertility of plants carrying *pKTN1*::*elk5-1D* matches the *elk5-1D* heterozygous phenotype while overexpressing plants have a sterile phenotype like *elk5-1D* (Figure 5B). This indicates that *elk5-1D* is a dominant mutant and exhibits a dose effect.

### 2.6. Aberrant Embryo Development and Twin Embryos in ktn1 Mutants

**Figure 6:**
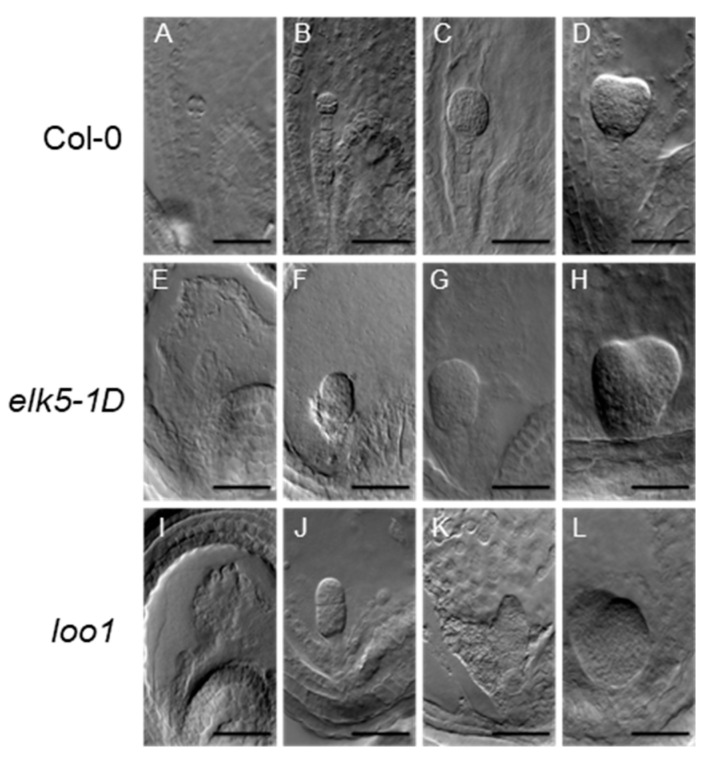
Aberrant embryo development in *elk5-1D* and *loo1* mutants. Col-0 embryos at different developmental stages, including 2 to 4 cells (**A**), octant (**B**), globular (**C**), and heart-shaped (**D**). Embryos of the *elk5-1D* and *loo1* mutant at the early globular stage (**E**,**F**,**I**,**J**), globular stage (**G**), and heart shape stage (**H**,**L**). Developmental arrest in the *loo1* mutant embryo (**K**). Scale bar = 50 μm.

**Figure 7:**
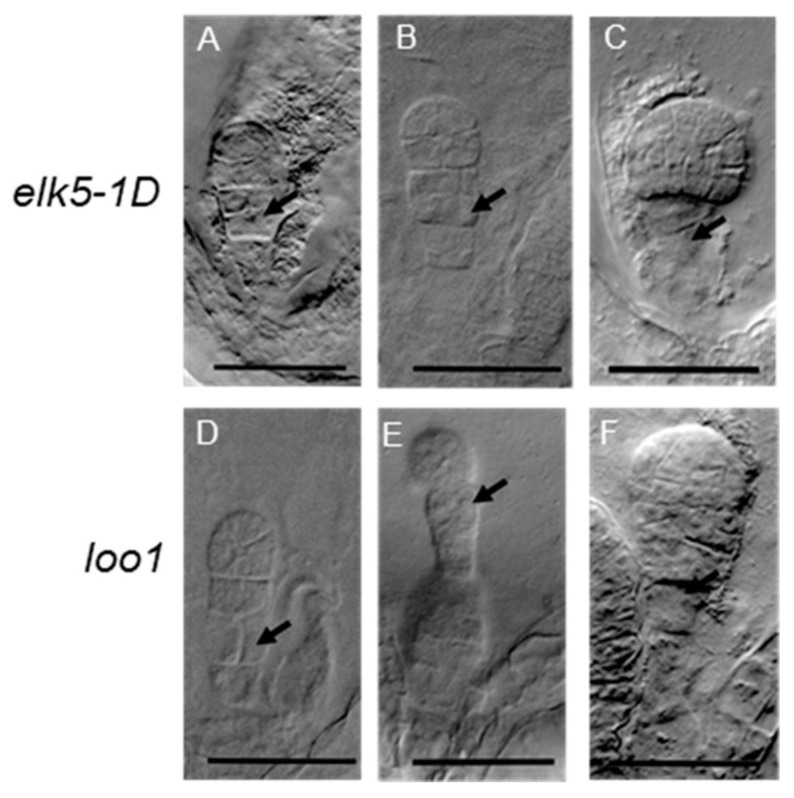
Abnormal cell division in the suspensor. Transverse cell division occurs in the hypophysis of *elk5-1D* (**A**–**C**) and *loo1* (**D**,**F**) as indicated by the arrow. The suspensor of *loo1* abnormally differentiates into a proembryo (**E**). Scale bar = 50 μm.

To further investigate seed development in *ktn1* mutants, DIC microscopy was used on dissection seeds of mutant and wild types. In particular, aberrant cell division orientations were observed in the proembryo and suspensor cells of the *elk5-1D* and *loo1* mutants (Figure 6 and Figure 7). Furthermore, in cases that exhibited greater severity, the morphology of the proper embryo markedly deviated from its typical spherical configuration (Figure 6F,G,J). Such irregular cellular proliferation in the early stages of embryogenesis ultimately resulted in the emergence of abnormal embryos during later growth phases (Figure 6H,K,L) compared to Col-0 (Figure 6A–D).

**Figure 8:**
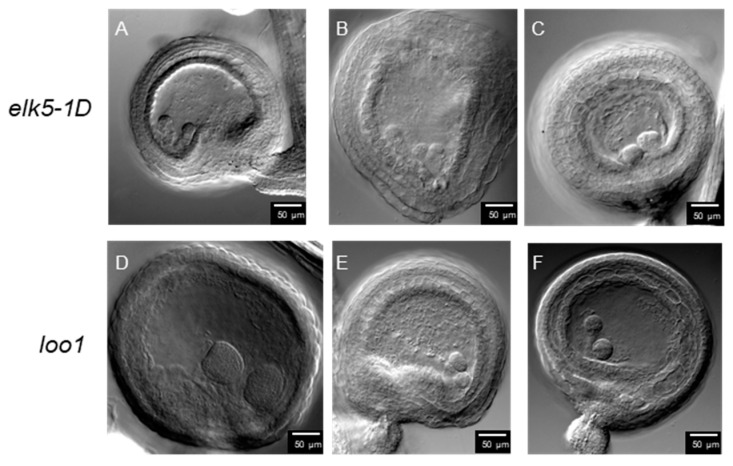
Twin embryos in *elk5-1D* and *loo1*. There are two embryos within *elk5-1D* (**A**–**C**) and *loo1* (**D**–**F**) seeds. Scale bar = 50 μm.

Surprisingly, our observations revealed twin embryos arranged side by side within the fertilized ovules of *ktn1* mutants (Figure 8). We conducted a survey of over a thousand ovules, finding twin-embryo frequencies of 0.62% in *elk5-1D* and 0.58% in *loo1*, while no instances were observed in the wild type (Table 1).

## 3. Discussion

The precise regulation of microtubule dynamics is crucial to ensure optimal cellular performance in a variety of fundamental biological processes. The exact control over microtubule abundance, the structural cohesion of their networks, and the dynamics of their assembly and disassembly are central to the successful realization of critical cellular functions such as mitosis, cellular differentiation, and migration activities [32]. Previous research has underscored the importance of the KTN1 protein in the gametogenesis of male and female individuals in various animal species [33,34,35]. However, the explicit role of KTN1 in the development of plant gametophytes and seed formation remains to be distinctly elucidated in the current body of research. In this investigation, we have identified two new mutated alleles of the *KTN1* gene, *elk5-1D* and *loo1*, each demonstrating disruptions in embryogenesis and seed development. These genetic variations are remarkable because of their link to an atypical phenotype, marked by the formation of twin embryos. This observation cautiously points towards a more profound understanding of the complex genetic mechanisms underlying plant embryogenesis. Furthermore, it opens avenues for future exploration of the potential contributions of the *KTN1* gene to plant morphology and reproductive strategies.

The fertility of the *elk5-1D* and *loo1* mutants is compromised, with sterility attributed to defects in the development of female gametophytes, while male gametophyte activity remains unaffected (Figure 2). Previous reports have indicated abnormal embryo sac morphology and significant changes in nuclear number within the embryo sac following *KTN1* mutation [30], potentially implicating differential developmental processes between male and female gametophytes. The development of the female gametophyte is a biphasic process. The initial phase, megasporogenesis, involves the conversion of a somatic cell in the nucleus of an incipient ovule into a megaspore mother cell (MMC) prepared for meiosis. Subsequent to megasporogenesis, there is megagametogenesis. During megagametogenesis, the functional megaspore (FM) undergoes a tripartite series of mitotic divisions, resulting in the formation of the embryo sac, or the female gametophyte [36,37]. The MMC undergoes meiosis, resulting in four megaspores; however, in the majority of angiosperm species, only a single megaspore survives and evolves into FM [38,39]. Asymmetric cell divisions and positional effects are common occurrences during the development of female gametophytes [31,40]. Abnormal division patterns observed in *ktn1* mutants suggest disrupted asymmetrical cell division (Figure 7). Compared to the hooked shape of wild-type embryo sacs, *ktn1* embryo sacs display a round morphology, which can cause faulty positional signals within the embryo sac and subsequent developmental defects [30,40]. In contrast, male gametophyte development is predominantly symmetrical; thus, the activity of *ktn1* mutants in male gametophytes remains largely unaffected. Furthermore, the number of male gametophytes is significantly greater than that of female gametophytes, providing greater tolerance to defective male gametophytes.

A base substitution (C to T) in *elk5-1D* leads to the replacement of Ser22 with phenylalanine in the C-terminal region (Figure 4B). The C-terminus of the KTN1 protein plays a crucial role in the assembly of the hexamer, and the final four amino acid residues (FGSA) at the C-terminus are highly conserved (Figure S1) [41]. In *Caenorhabditis elegans*, mutations of Phe469 and Gly470 at this site impair the ATPase activity and microtubule severing function of the katanin protein [41,42,43]. Serine, threonine, and tyrosine are common target residues of phosphorylation [44]. The mutation of this conserved serine residue in *elk5-1D* gives KTN1 a dominant function, leading us to predict that the last 4 aa peptide may be a phosphorylation site. Phosphorylation is a crucial post-translational modification of proteins, regulating protein conformation, stability, transport, and interactions while also modulating cellular dynamics and plasticity [45]. The dominant *elk5-1D* mutation substitutes Ser522 with phenylalanine, which could impact hexamer formation or impair ATPase activity and microtubule severing function due to loss of the potential phosphorylation of Ser522. Consequently, in *elk5-1D* heterozygous mutants, coexisting wild-type and mutant KTN1 proteins may interact together to form oligomers but are unable to form hexamers completely, or they may form heterohexamers but cannot perform microtubule severing. In contrast, the C-terminal deletion in *loo1* loses the oligomerization domain, potentially preventing mutated proteins from joining KTN1 hexamers formation without affecting the function of wild-type KTN1 hexamers. Thus, functional hexamers can still be formed by the wild-type KTN1 protein in *loo1* heterozygous mutants, explaining why *elk5-1D* is a dominant mutation while *loo1* is recessive.

We have identified a previously unreported phenomenon in *KTN1* mutants: the emergence of a twin embryo phenotype (Figure 8). Within the primordial structures of the *ktn1* ovule, cells adjacent to the MMC were found to exhibit notable enlargement and partially express MMC identity markers [31]. Therefore, in *ktn1* mutants, each ovule may give rise to more than just one embryo sac. Each sac harbors a complete set of egg cells and central cells, each independently capable of undergoing fertilization. This could potentially lead to the development of twin embryos. Additionally, the ultimate positioning of nuclei within the female gametophyte plays a pivotal role in determining the cell fate of the egg apparatus. Artificial manipulation of nuclear positioning, achieved through disturbance of the actin cytoskeleton, has the potential to re-calibrate the cell fate of the egg apparatus [46]. The *ktn1* embryo sacs exhibit a round morphology, which may lead to faulty positional signaling within the embryo sac, potentially enabling the synergid cells to acquire fertilization capacity. It is plausible that in *knt1* mutants, synergids may gain fertilization capacity, potentially contributing to the development of twin embryos. In early seed development, severing the connection between the suspensor and the embryo allows suspensor cells to potentially develop into secondary embryos [47]. In *ktn1* mutants, the suspensor exhibits abnormal division (Figure 7). Furthermore, the twin embryo phenotype observed in the *elk5-1D* and *loo1* mutants may also originate from atypical developmental processes in the suspensor. Investigating the functional role of KTN1 during female gametogenesis presents a compelling avenue for future study.

## 4. Materials and Methods

### 4.1. Plant Materials and Growth Conditions

*Arabidopsis thaliana* of the wild-type Columbia (Col-0) and Landsberg *erecta* (Ler) ecotypes, along with *elk5-1D* (CS3939) and *loo1* mutants, were utilized in this study. The *loo1* mutant was recovered from a mutant population (CS23153) obtained from the Arabidopsis Biological Resource Center (ABRC, Columbus, OH, USA). These lines were generated by transforming of WT *A. thaliana* (Col) plants with the pSKI15 with 4x 35S enhancers [48]. This *loo1*, selected due to its vegetative growth phenotype similar to *erecta* mutants, has not been previously reported. The seeds of both Col-0 and the mutants underwent surface sterilization, followed by a stratification process lasting three days at 4 °C. Post-stratification, they were sown on 1/2 Murashige and Skoog (MS) plates, supplemented with 1.0% sucrose (wt/vol). These plates were then placed in a growth chamber, maintained under a 16 h light/8 h dark photoperiod. After initial growth, the seedlings were transferred to an environmental chamber set at a constant temperature of 22 °C and the same photoperiodic conditions.

### 4.2. Alexander Staining

To assess pollen viability, stamens extracted from floral buds were carefully arranged on a microscope slide. Subsequently, a measured quantity of Alexander stain Solution (Solarbio) was applied. The stained pollen grains were then examined using an OLYMPUS dissecting microscope equipped with a digital camera. Following this staining process, viable pollen appeared purple, whereas non-viable, or dead pollen, was distinguished by its green coloration.

### 4.3. Phenotyping of the Mutant Lines

In the process of morphological phenotyping, a range of plant structures, including seedlings, rosette leaves, individual leaves, siliques, main inflorescence stems, inflorescences, and flowers, were systematically placed on a flat surface and captured using a digital camera. For seed morphology assessment, images taken with an OLYMPUS dissecting microscope were analyzed with ImageJ 1.54g software (https://imagej.net/ij/download.html (accessed on 8 November 2023)) to determine seed area, length, and width. The analysis of silique and seed development entailed the careful dissection of siliques with fine tweezers, followed by microscopic photography to document the seeds and any aborted ovules.

### 4.4. Genetic Mapping

The *KTN1* gene was successfully isolated by employing a map-based cloning strategy. An F2 segregating family originated from a cross between an *elk5-1D* and *loo1* homozygote in the Columbia background and a wild-type Landsberg *erecta*. Initial rough mapping utilized SSR markers, analyzing DNA from *elk5-1D* and *loo1* F2 mutants of the mapping population. For finer resolution mapping, DNA polymorphisms from the Cereon Genomics database (https://www.arabidopsis.org/browse/Cereon/index.jsp (accessed on 5 March 2023)) facilitated the generation of SSLP markers. The primers applied for this fine mapping included CIW1 (5′-AGGTTTTATTGCTTTTCACA-3′ and 5′-CTTTCAAAAGCACATCACA-3′), NGA128 (5′-ATCTTGAAACCTTTAGGGAGGG-3′ and 5′-GGTCTGTTGATGTCGTAAGTCG-3′), NF5I14 (5′-GGCATCACAGTTCTGATTCC-3′ and 5′-CTGCCTGAAATTGTCGAAAC-3′), and NGA111 (5′-TGTTTTTTAGGACAAATGGCG-3′ and 5′-CTCCAGTTGGAAGCTAAAGGG-3′). DNAs from F2 *elk5-1D* and *loo1* mutants from the mapping population were analyzed using these primers. For the identification of mutations in *elk5-1D* and *loo1* alleles, the *KTN1* coding region was PCR-amplified from both wild-type and mutant plant DNAs using Taq polymerase (YEASEN). Subsequent to purification, these PCR products were directly sequenced.

### 4.5. Plasmid Construction and Generation of Transgenic Plant Generation

The construction of the *pKTN1*::*elk5-1D* vector involved PCR amplification of the mutated *elk5-1D* allele, along with its native promoter and terminator, using genomic DNA and specific primers (Forward: 5′-gtggcggccgctctagaaGGTTTGATGGACCTGCAGAT-3′, Reverse: 5′-aattcctgcagcccggggCTTCGCCGTCTCTACGGAAA-3′). The amplified products were subsequently purified and inserted into the *Spe*I and *Bam*HI sites of the *pSSR100* vector, utilizing the Hieff Clone^®^ Plus One Step Cloning Kit, to generate the *pKTN1*::*elk5-1D* construct. For the overexpression, the mutated *elk5-1D* allele was amplified using a different set of primers (Forward: 5′-catttcatttggagaggatATGGTGGGAAGTAGTAATTCGT-3′, Reverse: 5′-aattcctgcagcccggggCTTCGCCGTCTCTACGGAAA-3′). The products of this amplification were purified and cloned into the *EcoR*V and *Hind*III sites of the *pSSR100*-*p35S*::*Clover* vector, employing the Hieff Clone^®^ Plus One Step Cloning Kit, to generate the *p35S*::*elk5-1D* vector. This plasmid was then used to transform *Agrobacterium tumefaciens* strain GV3101. *Arabidopsis thaliana* Col-0 plants were subsequently transformed with this construct using the well-established floral dip method.

### 4.6. Reverse-Transcription PCR (RT-PCR) Analysis

The synthesis of first-strand DNA was conducted using 1 µg of total RNA from *A. thaliana*, utilizing the HiScript II 1st Strand cDNA Synthesis Kit (Vazyme) with an oligo (dT) primer. To amplify the *ktn1* transcripts in *loo1*, primers specifically designed were used: forward primer 5′-CTGCTTGAGGAGGCAGTTGTC-3′ and reverse primer 5′-GTTTAATTAAGCAGATCCAAAC-3′. These primers were designed to yield a 793 bp fragment from cDNA templates and a 1249 bp fragment from genomic DNA templates. The PCR reactions were performed in a 50 µL volume, using 1 µL of the RT reaction mixture as the template. The cycling conditions included an initial denaturation at 94 °C for 5 min, followed by 33 cycles, each consisting of 30 s at 94 °C, 30 s at 58 °C, and 30 s at 72 °C. The RT-PCR products were analyzed on a 1% (*w*/*v*) agarose gel, with visualization via ethidium bromide staining. To confirm the *ktn1* transcripts in *loo1*, we sequenced the PCR products and aligned them to the *KTN1* genomic sequence.

### 4.7. Light Microscopy

To investigate female gametophyte development, we selectively used emasculated flowers and developing buds at precise developmental stages. The preparation of samples and observations under differential interference contrast (DIC) microscopy were conducted as previously outlined [49]. To investigate the development of the endosperm and embryo, siliques ranging from 1 to 5 days post-fertilization were collected. These were meticulously opened along both sides of the pistil replum using precise tweezers to expose the ovules. The samples were fixed for one hour and washed according to the protocol previously described. The ovules were then carefully separated from the placenta and submerged in 60 μL of a clearing solution (comprising chloral hydrate, water, and glycerol in an 8:2:1 ratio) within a 0.5 mL tube. They were left to clarify at room temperature for two hours. Post-incubation, the clarified ovules were mounted on a glass slide, covered with a coverslip, and examined using the differential interference contrast (DIC) optics of an Olympus microscope equipped with a digital camera.

## 5. Conclusions

In this study, we identified two novel *KTN1* gene variants, *elk5-1D* and *loo1*, which exhibit significant organ shortening, similar to previously reported *ktn1* mutants. We found that infertility in both *elk5-1D* and *loo1* is due to abnormalities in the development of the female gametophyte and shortened filaments. Our findings indicate that *elk5-1D* is a dominant mutation with a dosage effect, potentially due to a serine-to-phenylalanine substitution. Further observations of early seed development in *elk5-1D* and *loo1* revealed irregular zygotic divisions and a rare occurrence of twin embryos, less than 1% frequency. Overall, these results underscore the critical role of *KTN1* in early embryonic development and its impact on cell fate determination.

## Figures and Tables

**Table 1:** Incidence rate of twin embryos in *elk5-1D* and *loo1*.

	No. of Fertilized Ovules	No. of Twin Embryos	Percentage of Twin Embryos
Col-0	1035	0	0
*elk5-1D*	1126	7	0.62%
*loo1*	1382	8	0.58%

## Data Availability

The authors confirm that the data supporting the findings of this study are available within the article.

## Supplementary Materials

The following supporting information can be downloaded at https://www.mdpi.com/article/10.3390/plants13131824/s1, Figure S1: Sequence alignment of KTN1 homologs. The blue line represents the four conserved amino acids at the C-terminus. The red pentagon indicates the serine that is replaced in *elk5-1D*.
