# Supplementary figures and images for: Twin Embryos in Arabidopsis thaliana KATANIN 1 Mutants
Source: Plants (Basel). 2024 Jul 3;13(13):1824. doi: 10.3390/plants13131824 (PMC11244573; doi:10.3390/plants13131824)

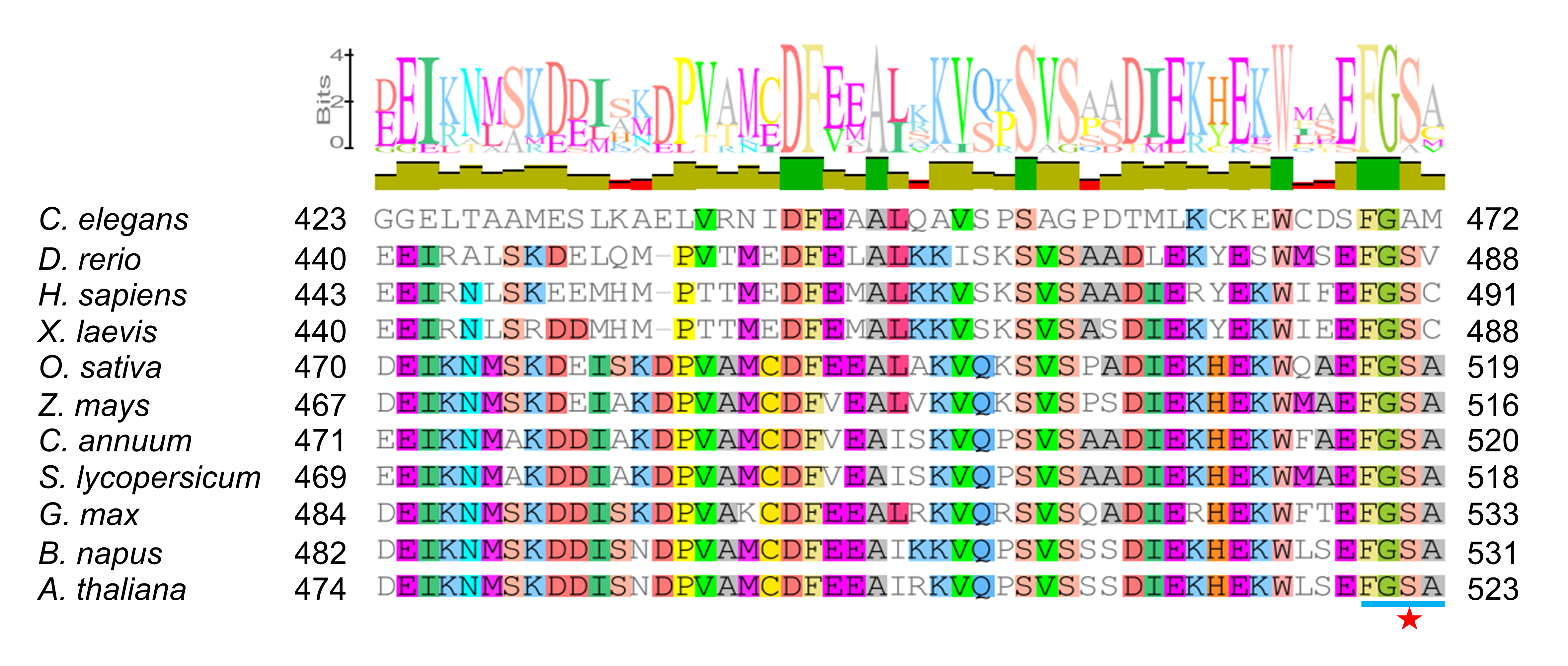

Supplement: Supplementary file 1 [file plants-13-01824-s001.zip › Figure S1.tif]
